# Seasonal Variations in Testicular Torsion: A Retrospective Study

**DOI:** 10.7759/cureus.76508

**Published:** 2024-12-28

**Authors:** Liqaa Raffee, Ma'moun Bani Ali, Khaled Alawneh, Nour Negresh, Hasan Alawneh, Ali Al-Shatnawi, Retaj Alawneh

**Affiliations:** 1 Department of Accident and Emergency Medicine, Jordan University of Science and Technology, Irbid, JOR; 2 Department of Emergency Medicine, King Abdullah University Hospital, Irbid, JOR; 3 Department of Diagnostic Radiology and Nuclear Medicine, Jordan University of Science and Technology, Irbid, JOR; 4 Division of Emergency, Department of Special Surgery, Faculty of Medicine, Al-Balqa Applied University, Al-Salt, JOR; 5 Department of Medical Engineering, Cardiff University School of Engineering, Wales, GBR; 6 Department of Internal Medicine, Jordanian Royal Medical Services, Amman, JOR; 7 Department of Emergency Medicine, Jordan University of Science and Technology, Irbid, JOR

**Keywords:** age distribution, doppler ultrasound, seasonal variation, signs and symptoms, testicular torsion

## Abstract

Background: Testicular torsion (TT) is a urological emergency requiring prompt intervention to prevent irreversible damage to the testicle.

Objective: This study aims to assess trends in men's TT referrals, diagnostic evaluation through Doppler sonography (DS) scan, symptoms before surgery, orchidectomy rates, and TT laterality in relation to age and seasons of the year.

Methods: This observational retrospective cohort study included all patients treated for TT at King Abdullah University Hospital between 2009 and 2021. Patients underwent DS, scrotal exploration, orchidopexy, or orchidectomy. Data collected encompassed patient demographics, admission date, duration of symptoms, laterality, and surgical intervention.

Results: Our study consisted of 308 TT patients between the ages of 10 and 33. Approximately 194 (63%) patients who were taken to the hospital for an average of 47 hours got an orchidectomy. Although the majority of patients received orchidopexy within 72 hours of detorsion and orchidopexy, five patients (3.6%) required reperfusion following detorsion and orchidopexy. A total of 112 individuals (36.4%) underwent bilateral orchidopexy, which lasted anywhere from 9 to 26 hours, whereas 194 (63%) patients underwent orchidectomy, which lasted anything from 40 to 264 hours (p < 0.001). The prevalence of right-sided TT increases by 6% per year with age (p = 0.047).

Conclusions: This study found a high association between the amount of recoverable testicular tissue and the duration of symptoms before surgery. Even after 72 hours, it is still possible to salvage the testicles, even though the success rate reduces from 75% within 24 hours to 3.6% beyond that. Additionally, elderly individuals are more likely to experience right-sided torsion. Seasonal variation was evident, with the highest incidence of TT occurring during winter months, suggesting potential environmental or physiological triggers.

## Introduction

Torsion of the testicles is a urological emergency. The testicular blood supply is shut off by the twiddling and rotation of the spermatic cord. During a twist, the testicle's blood supply is cut off, resulting in intense inflammation and pain. In the event of testicular torsion (TT), immediate surgery is essential to save the testicle (also known as testicle detorsion). Long-term TT can cause irreversible damage to the testicle, necessitating its removal. The angle of twist varies between 180° and 720°. The testicles are damaged at a steady pace according to the level of twisting. Within four to six hours, 90% of testes can be salvaged. It is unlikely that a testicle can be saved if left untreated for more than 24 hours.

The homolateral testicle is deprived of blood flow due to an aberrant twisting of the spermatic cord. To maintain the testicle and ensure future fertility, early diagnosis and treatment are essential [1-3]. According to new research, patients with acute TT may have a better prognosis than previously believed. Patients who arrive late have a greater likelihood of undergoing an orchiectomy. Some studies have indicated a favorable association between cold weather and the incidence of TT, whereas others have found no correlation [4].

In 2015, the following two-week period of observations generated this study: left-sided TT children under the age of 16 were frequently brought to the Department of Emergency at King Abdullah University Hospital (KAUH), where testicular loss was prevalent (orchidectomy). In cases of acute scrotum, patient referrals were delayed due to lack of expertise, wrong diagnosis, and misdiagnosis.

We aimed to assess annual trends in the number of patients referred to the hospital for radiology or surgery, including cases involving manual detorsion, testicular salvage, or orchidectomy. Additionally, our investigation sought to explore potential seasonal patterns of TT and examine the age and side of disease presentation.

## Materials and methods

Study design and data collection

This study was conducted at the Emergency Department, KAUH, Irbid, Jordan. Amman, the capital city of Jordan, is home to the Blinded, which has the country's principal tertiary care hospital. As part of a retrospective and descriptive study, researchers utilized the medical records of patients from 2009 to 2021. The Human Research Ethics Committee gave the probe permission to launch before it ever left the ground (48/137/2021, dated January 14, 2021). Additionally, the Department Head and Chief Executive Officer of Blinded granted approval for the use of the hospital's medical records. This study was conducted in accordance with the 1964 Helsinki Declaration.

All patients with TT treated at KAUH between 2009 and 2021 with Doppler sonography (DS) and scrotal exploration, orchidopexy, or orchidectomy were included in the study. Pediatric surgeons handle patients under the age of 10 in accordance with hospital policy and interdepartmental agreements. It was determined that deidentified patient data should be used during testing to protect their privacy.

A preliminary investigation was done using theater data. An examination of the scrotum, an orchidectomy, and an orchidopexy were used as keywords. Only patients with TT were examined in this study. Three hundred fifty-one sets of medical records were collected in total. In the medical records of patients who had undergone surgery for an undescended testis or a penetrating scrotal injury, only 308 instances of TT were identified. Data collection included the patient's age, month of admission, year of admission, reason of torsion, side of pathology, duration of symptoms before admission, manual detorsion, time from admission to surgery, and operation type (orchidopexy or orchidectomy).

On the first page of the blue file, control chart, and nursing progress report, the preadmission symptoms and time of admission are listed. The period between the patient's admission and surgery was recorded in the anesthetic chart or operating room records. The Excel file (Microsoft Corporation, Redmond, WA) containing the unique identifier for each item was only accessible to researchers.

Statistical analysis

The data were analyzed using version 26 of the Stata Statistical Software (StataCorp, College Station, TX). Because the time frame data were not normally distributed, we used medians and interquartile ranges (IQRs) instead of standard deviations (SDs). Categorical data are summarized using frequencies and percentages, while continuous data are analyzed and presented using the median, IQR, and mean (SD). Researchers utilized the chi-square test to investigate the seasonality and laterality of the disease. The Wilcoxon rank-sum test was used to find a difference in medians between the two groups. We defined statistical significance for the purposes of this study (p < 0.05).

## Results

In this study, 308 individuals with TT were examined. Participants ranged in age from 10 to 33, with a mean age of 17.20 ± 4.13 years. Two men in the research had their orchiectomy because atrophies caused by months of testicular discomfort following contralateral orchidopexy caused their testicles to atrophy (cause for consultation). The opposite side of the twist began seven days after bilateral torsion simultaneously (one year apart).

**Figure 1:**
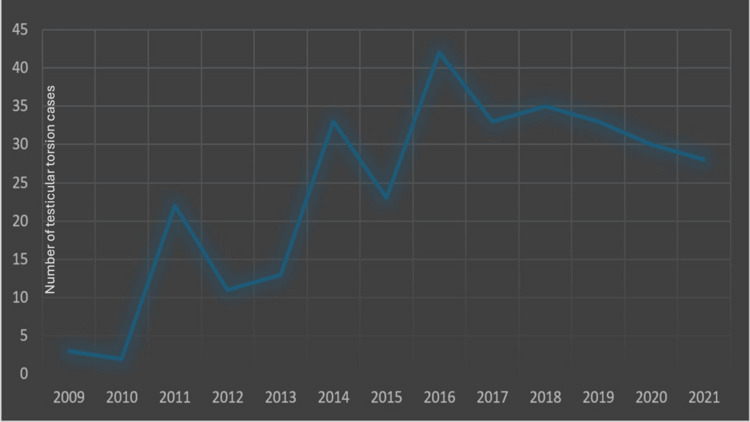
Annual distribution of TT cases from 2009 to 2021 TT: testicular torsion

As depicted in Figure 1, the number of referrals climbed progressively from a low of two in 2009 to a high of 42 in 2013. Throughout the 13 years of the study, an average of 24 patients per year were sent to the research team. The bulk of the population was above the age of 16 (57.1%), while just 132 (42.8%) were under the age of 16. During the winter months, the greatest number of people seek therapy (Table 1). More than 296 (96%) instances were the result of spontaneous causes, whereas only 12 (4%) were the result of trauma. Thirty-six hours (10-168) passed from symptom onset to hospital admission, 6 hours (3-48) from hospital admission to surgery, and 47 hours (15-216) from surgery to symptom onset for each patient.

**Table 1:** Demographic characteristics and distribution of patients across seasons and study years Data are presented as n (%) unless stated otherwise SD: standard deviation

Demographics	Overall, n (%)
Age in years (mean ± SD)	17.20 ± 4.13
Number of patients less than 16 years	132 (42.8%)
Number of patients more than 16 years	176 (57.1%)
Summer	61 (19.8%)
Autumn	79 (25.6%)
Winter	92 (29.8%)
Spring	76 (24.6%)
Year 2009	3 (0.97%)
Year 2010	2 (0.64%)
Year 2011	22 (7.14%)
Year 2012	11 (3.5%)
Year 2013	13 (4.2%)
Year 2014	33 (10.7%)
Year 2015	23 (7.4%)
Year 2016	42 (13.6%)
Year 2017	33 (10.4%)
Year 2018	35 (11.3%)
Year 2019	33 (10.4%)
Year 2020	30 (9.7%)
Year 2021	28 (9.09%)

Patients who come into the operating room within 72 hours after the onset of symptoms have a significant reduction in delays. One hundred fifty-eight (51.6%) of the 308 study participants had left-sided torsional damage, 148 (48.6%) had right-sided torsional injury, and one patient (0.3%) experienced bilateral torsional injury: 12 hours (7-24 hours) to 3 hours (2-5 hours), and 16 hours (10-28 hours) to 10 hours (2-5 hours). The average age for males with left TT was 16.73 years, whereas the mean age for those with right TT was 17.66 years. Only 76 (47.7%) of those under 16 and 82 (51.5%) of those over 16 had left-sided torsion, according to (Table 2). There was a statistically insignificant trend toward right-sided TT in patients older than 16 years.

**Table 2:** Correlation of age with sides of TT CI: confidence interval; TT: testicular torsion

Characteristics	Left (n = 159)	Right (n = 149)	Total (n = 308)	Odd ratio (95% CI)	p value
Age in years	16.73 ± 3.84	17.66 ± 4.36	17.20 ± 4.12	1.05	0.046
Age <16 years	76 (47.7%)	55 (36.9%)	132 (42.8%)	1.00	0.079
Age >16 years	82 (51.5%)	94 (63.0%)	175 (56.8%)	1.49	

**Figure 2:**
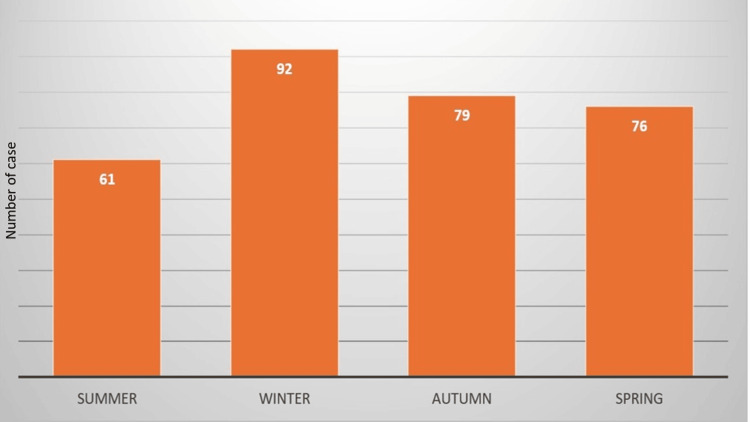
Seasonal distribution of TT cases TT: testicular torsion

Table 3and Figure 2demonstrate the number of patients who are presented to the department during each season.

**Table 3:** Age groups and seasons of the year Data are presented as n (%) unless stated otherwise

Characteristics	Summer	Autumn	Winter	Spring	Total
Age <16 years	30 (22.7%)	32 (24.2%)	35 (26.5%)	35 (26.5%)	132 (100%)
Age >16 years	31 (17.6%)	47 (26.7%)	57 (32.3%)	41 (23.2%)	176 (100%)
Total number of patients	61 (19.8%)	79 (25.6%)	92 (29.8%)	76 (24.6%)	308 (100%)

One hundred twelve (36.4%) of the 308 study participants had bilateral orchidopexy, 194 (63%) had an orchidectomy, and two (0.65%) had a contralateral orchidopexy because the infected diseased testis had atrophied. The majority of patients 82 (74.55%) underwent salvage surgery (or orchidopexy), whereas the remaining 28 (25.45%) underwent orchidectomy. Patients who presented within 24 hours of the study's initiation had a 35.7% likelihood of participation.

In patients who underwent surgery within 72 hours, the orchidectomy rate increased to 95%, but the orchidopexy rate decreased to 3.6% (Table 4). Only 13 hours (9-26 hours) of symptoms were encountered by people who underwent bilateral orchidopexy, whereas individuals who underwent orchidectomy experienced 144 hours (40-264 hours) of discomfort (p < 0.001). Before hospitalization and surgery, patients who had bilateral orchidopexy experienced a much slower onset of symptoms than those who had undergone orchidectomy (p < 0.001). Three hours after the onset of symptoms, the initial manual detorsion was conducted, whereas the ultimate manual detorsion was performed 48 hours later. Patients who underwent manual detorsion within 48 hours of presenting were 11.5% more likely to undergo bilateral orchidopexy than orchidectomy.

**Table 4:** Onset of symptoms after surgery Data are presented as n (%) unless stated otherwise

Types of surgery	<24 hours	>24 to <72 hours	>72 hours	Total number of patients
Bilateral orchidopexy	82 (74.55%)	25 (41.00%)	5 (3.6%)	112 (36.4%)
Orchidectomy	28 (25.45%)	36 (59.00%)	130 (95%)	194 (63%)
Contralateral orchidopexy	0 (0%)	0 (0%)	2 (1.4%)	2 (0.6%)
Total number of patients	110 (100%)	61 (100%)	137 (100%)	308 (100%)

## Discussion

TT is a surgical emergency and exists due to the possibility of permanent testicular ischemia. Ischemia can occur within six hours, and the likelihood of preserving the testis decreases during the following 48 hours [5-7]. When the color Doppler demonstrates no intratesticular blood flow, torsion is caused by a rotation of more than 360°. A few minutes of discomfort that resolves on its own is indicative of intermittent torsion. Even when torsion is less than 360° in partial or whole cases, intratesticular perfusion can still be observed [6]. Discomfort does not vanish by itself.

In addition, the patient may be compelled to awaken due to the pain. TT is characterized by scrotal pain, edema, erythema, nausea, and vomiting. Any attempt at testicular detorsion will fail due to the absence of precise diagnostic information. Imaging and other diagnostic techniques that increase diagnostic time must be discontinued. This effect is known as the Doppler effect; DS can detect a positive whirlpool sign or lack of testicular flow when there is a testicular twist [8]. Imaging can still be performed if the presentation is delayed by more than 72 hours.

The prevalence of orchidopexy and orchidectomy has been documented in numerous international studies. Between 1992 and 2010, there were 21,289 cases of TT in the United States. The annual incidence rate was determined to be 1.4:100,000 [9]. According to the Taiwanese National Health Insurance Research Database, only 25.6% of Asian men under the age of 25 who were diagnosed with TT underwent orchiectomies, and 74.4% underwent orchiopexies between 1997 and 2010. The prevalence of TT is estimated to be 3.5 per 100,000 patients a year [10]. TT was found in 89 of the 173 scrotal exams performed in England between 1998 and 2008, and 16 of those cases necessitated an orchidectomy because of the delayed presentation [11]. From August 1995 to September 2007, all testicular and paratesticular specimens in Australia were examined for their pathology. The clinical data were collected from a computerized database; 11.2% of study participants had TT, with 68% of those cases occurring on the left [12]. Zimbabwe enrolled about 90 African patients with TT between 1987 and 1991 (between two months and 32 years). Compared to the 71% who presented late (>72 hours), just 39% presented within 72 hours, with a seven-day average delay. The orchiectomy rate was 64%, while the testicular salvage rate was 36% [13]. In Nigeria, on the opposite side of Africa, Udeh discovered a stunning orchidopexy rate of 75% [14].

According to this study, the average age of referrals is increasing, with a range of 10-33 years and a mean of 17.20 ± 4.13 years. Due to shoddy documentation and illegible handwriting, it was impossible to identify many records, especially those created between 2009 and 2013. As a result, we are unable to discuss this particular pattern in greater detail. Despite the recognition of pediatric urology as a specialty, the number of referrals for TT increased from three in 2009 to 42 in 2016.

The majority of patients in our study were hospitalized between 10 and 168 hours (36 hours) following their initial symptoms, which accounted for the 63% orchidectomy rate in our study. Before admission, delays are most prevalent. It is noteworthy that the orchidectomy rate in this study is higher than those discovered in Taiwan (25.6%) and England (18%) [15]. There are numerous similarities between the findings of the Zimbabwean study and those of this investigation. According to Rampaul and Hosking, delayed hospitalization was the leading cause of testicular loss in patients with TT [16]. The patient's outcome was unaffected by a few hospital delays.

Peeraully et al. discovered that patients who presented directly to their emergency department had a 23% lower chance of undergoing an orchidectomy than those who were referred from primary care centers (43%) or transferred to other hospitals (50%) [17]. Our tertiary hospital receives a significant number of referrals and transfers from primary care facilities. Our clinic received patients nationwide, making it hard to calculate an incidence rate.

In men with TT, the outcomes of scrotal examination are strongly reliant on the duration and degree of symptoms. According to a study by Feng et al., testicles can no longer be saved after 13.5 hours of symptom onset [18]. After 72 hours, our research revealed that testicular salvage was still possible in cases involving intermittent torsion or in the condition of partial blood flow preservation, and surgical notes confirmed that the testicles had been completely torn. After surgery, they may have experienced intermittent torsion or torsion detorsion syndrome despite having entire torsion.

If a patient is assessed within 24 hours of the onset of symptoms, his likelihood of testicle preservation increases to as high as 75%, compared to 25% with an orchidectomy. Table 4 shows that patients who arrived after 72 hours had a 95% orchidectomy rate compared to a 3.6% orchidopexy rate. After detorsion and wrapping the testis in a warm, wet surgical swab, the testis' vitality is frequently confirmed by a shift in color from blue to pinkish. Following detorsion in the operating room, patients who displayed symptoms of reperfusion received orchidopexy. Five patients (3.6%) who presented after 72 hours underwent orchidopexy between 3 and 34 days after the beginning of the study (about five weeks). One year after investigating 16 cases of TT and performing surgery on two of them within 72 hours, Sato et al. discovered no signs of testicular atrophy [19]. Testicular salvage surgery has a higher survival rate for those who arrive at the hospital sooner.

In the orchidopexy group, the median time from symptom onset and surgery was 13 hours (9-26 hours), but in the orchidectomy group, it was 144 hours; the difference was statistically significant (p < 0.001). In other words, the median time between admission and surgery was shorter for the orchidopexy group, ranging from 24 to 72 hours, than for the orchidectomy group. According to English researchers, TT is a typical reason for treatment delays. Within an hour of developing symptoms, patients were brought to the operating room, and from that point on, the median time for patients who received orchidopexy was 5.5 hours compared to 42 hours for patients who underwent orchidectomy [16]. The participants in both therapy groups spent six hours a day together, according to a Croatian study [20]. In our investigation, the median lengths of both groups were longer than in any prior [21-23]. It may be important to conduct additional research on the level of awareness, distance and transportation obstacles, and logistical difficulties during referral or transfer to better comprehend these findings.

According to research by Korkes et al. and Pogorelic et al., the winter months appear to be the busiest season for new cases [9,20]. Table 3 shows that 32.3% were for individuals aged 16 and older during the winter, but this was not statistically significant (p = 0.480). The risk of TT increases as a result of a fall in temperature. This is due to the natural physiological response of the body to protect the testicles from the cold weather by moving the testicles up closer to the body by contracting the cremaster muscle. Excessive contraction usually leads to torsion and, eventually, death of the testicles [24].

In 28 of 32 patients (87.5%) who arrived within 48 hours, manual detorsion and testicular salvage or fixation surgery were highly associated. To minimize the duration of testicular ischemia, orchidopexy is performed after this treatment [25]. If a case of probable TT is reported within 72 hours, we accelerate the patient's hospitalization and discharge. If a patient is admitted within 72 hours of the onset of symptoms, physical detorsion by a surgical intervention is often necessary to prevent additional ischemia of the testis, particularly in circumstances involving severe trauma, such as gunshot or stab wounds.

As we predicted, no previous research has focused on the relationship between age and TT lateralization. Despite the lack of statistical significance, it indicates that patients aged 16 years are 1.5 times more likely to present with right-sided TT (p = 0.08). According to the same study, the likelihood of developing right-sided TT increased by 6% per year. Right-sided TT in elderly patients is unlikely to be clinically significant. The clinical implications of this observation may require additional investigation.

There was no electronic record-keeping system in the operating room and intensive care unit, and files could not be retrieved due to misspelled names, incorrectly entered hospital numbers, and illegible handwriting. It was unable to obtain follow-up notes for orchidopexy beyond 72 hours because outpatients have their own outpatient files. It is probable that preadmission delays were caused by one of the following: a lack of competency, a misdiagnosis, a concern about distance and transportation, or logistical concerns related to transfer preparations.

Our results should be interpreted with caution. First, the nature of the retrospective design may introduce selection biases. Second, being a single-center study limits the generalizability of findings to other populations or healthcare settings. Finally, the concept of the descriptive study utilized in this article weakens our findings. Future multicenter, prospective, and comparative studies are recommended to validate the results and ensure broader applicability across diverse contexts.

## Conclusions

TT is a surgical emergency that requires prompt diagnosis and intervention to preserve testicular viability and prevent orchidectomy. This study highlights significant delays in presentation and treatment, resulting in a high orchidectomy rate of 63%, particularly in cases presenting beyond 72 hours. Seasonal variations and left-sided predominance were observed, with winter showing the highest incidence. Despite advancements in pediatric urology and increased referrals, delayed presentation remains a critical challenge. Public awareness campaigns emphasizing the symptoms and urgency of TT, along with educational programs targeting primary care providers, can play a pivotal role in improving early recognition and timely referral. Enhanced access to healthcare services, streamlined referral pathways, and the use of telemedicine for remote consultations may also reduce delays and improve testicular salvage rates, ultimately decreasing the burden of preventable testicular loss.
